# Decoding the codon usage patterns in Y-domain region of hepatitis E viruses

**DOI:** 10.1186/s43141-022-00319-2

**Published:** 2022-04-11

**Authors:** Zoya Shafat, Anwar Ahmed, Mohammad K. Parvez, Shama Parveen

**Affiliations:** 1 Centre for Interdisciplinary Research in Basic Sciences, Jamia Millia Islamia, New Delhi, India; 2 Centre of Excellence in Biotechnology Research, College of Science, King Saud University, Riyadh, Saudi Arabia; 3 Department of Pharmacognosy, College of Pharmacy, King Saud University, Riyadh, Saudi Arabia

**Keywords:** YDR, Nucleotide composition, Codon usage bias, Mutation pressure, Natural selection

## Abstract

**Background:**

Hepatitis E virus (HEV) is a positive-sense RNA virus belonging to the family *Hepeviridae.* The genome of HEV is organized into three open-reading frames (ORFs): ORF1, ORF2, and ORF3. The ORF1 non-structural Y-domain region (YDR) has been demonstrated to play an important role in the HEV pathogenesis. The nucleotide composition, synonymous codon usage bias in conjunction with other factors influencing the viral YDR genes of HEV have not been studied. Codon usage represents a significant mechanism in establishing the host-pathogen relationship. The present study for the first time elucidates the detailed codon usage patterns of YDR among HEV and HEV-hosts (Human, Rabbit, Mongoose, Pig, Wild boar, Camel, Monkey).

**Results:**

The overall nucleotide composition revealed the abundance of C and U nucleotides in YDR genomes. The relative synonymous codon usage (RSCU) analysis indicated biasness towards C and U over A and G ended codons in HEV across all hosts. Codon frequency comparative analyses among HEV-hosts showed both similarities and discrepancies in usage of preferred codons encoding amino acids, which revealed that HEV codon preference neither completely differed nor completely showed similarity with its hosts. Thus, our results clearly indicated that the synonymous codon usage of HEV is a mixture of the two types of codon usage: coincidence and antagonism. Mutation pressure from virus and natural selection from host seems to be accountable for shaping the codon usage patterns in YDR. The study emphasised that the influence of compositional constraints, codon usage biasness, mutational alongside the selective forces were reflected in the occurrence of YDR codon usage patterns.

**Conclusions:**

Our study is the first in its kind to have reported the analysis of codon usage patterns on a total of seven different natural HEV hosts. Therefore, knowledge of preferred codons obtained from our study will not only augment our understanding towards molecular evolution but is also envisaged to provide insight into the efficient viral expression, viral adaptation, and host effects on the HEV YDR codon usage.

**Supplementary Information:**

The online version contains supplementary material available at 10.1186/s43141-022-00319-2.

## Background

Hepatitis E virus (HEV) is the cause of both epidemic and sporadic hepatitis cases in humans [1, 2]. HEV is a positive-sense, single-stranded RNA virus, belonging to the family *Hepeviridae*. The 7.2 kb genome of HEV, with short 5′ and 3′ non-coding regions (NCR), consists of three partially overlapping open reading frames (ORFs) [3]. The 5′ most ORF (ORF1) encodes the non-structural polyprotein which is organized into seven functional domains including the Y-domain region (YDR) [4, 5], 3' most ORF (ORF2) codes for the viral capsid protein [6, 7], and ORF3 encodes the phosphoprotein responsible for viral regulation [8–10]. The non-structural ORF1 Y-domain region (YDR) critical residues have been demonstrated to play critical role in the HEV life cycle [11].

HEV is segregated into four major genotypes (HEV-1 to HEV-4), out of which HEV-1 and HEV-2 infect humans, while HEV-3 and HEV-4 strains have an expanded range of hosts which includes humans, rabbits, wild boars, and pigs [12–25]. Studies have reported the isolation of other strains of HEV from specific hosts, such as HEV-5 and HEV-6 from wild boars in Japan [14, 15]. HEV-7 from dromedary camels [24] and HEV-8 from Bactrian camels [25]. The genetic code encompasses 64 codons, separated into 20 distinguishable groups. Each individual group, consists of one to six codons, encodes the same amino acid. Thus, each standard amino acid is often encoded by alternative codons belonging to the same group. These alternative codons are termed as “synonymous” codons. These synonymous codons differ not only between genomes but also within the same genome of the organisms/organism. This phenomenon is referred to as codon usage bias [26, 27] and has been well documented in many organisms including prokaryotes, eukaryotes, and viruses [28–33].

Previous reports on codon usage have determined various factors governing the codon usage patterns which include mutational pressure, translational selection, G + C content secondary structure of protein, selective transcription replication, hydrophilicity, and hydrophobicity of the protein and the external environment [28, 34–36]. Among these, compositional constraints under natural selection and mutational pressure are two major paradigms in shaping the codon usage patterns in organisms [37–39]. However, in viruses, mutational pressure rather than natural selection is found to be the major factor influencing codon usage variation [40–43].

As YDR indispensability in HEV pathogenesis has been demonstrated [11], thus, it is important to determine the distinctive genetic features that are prevalent in their genomes. Using an interdisciplinary systems biology approach, we attempted to explain the codon usage bias of HEV-hosts in conjunction with evolutionary forces (compositional, mutational, selection) accountable for shaping the YDR codon usage patterns. The present study is the first in its kind which have reported the detailed codon usage analysis on a total of 7 hosts in HEV YDR. Therefore, knowledge obtained from the presented study will not only augment our understanding towards molecular evolution but is also envisaged to provide insight into the efficient viral expression, viral adaptation and host effects on HEV [31, 44].

## Methods

### Heat map construction

The heat map was constructed using the online software tool Morpheus (https://software.broadinstitute.org/morpheus/documentation.html). Heat map is one of the most commonly used visualization in the science field because it allows us to find patterns in our data, compact a large amount of information into a small space, and are a natural representation of a matrix.

### Sequence data acquisition

The YDR sequences were accumulated from the National Centre for Biotechnology information (NCBI). The retrieved sequences were selected based on the following inclusion criteria: (a) The strain (GenBank Accession number: NC_001434.1) was used as reference strain; (b) sequences were included from different hosts encompassing human, rabbit, pig, mongoose, wild boar, camel, and monkey; (c) sequences from same or different regions at varying time intervals were considered to avoid repetition in analysis; and (d) sampling dates of the sequences were clearly stated. Accumulated sequences from NCBI were edited using the Bioedit v.7.2 sequence analysis software (http://bioedit.software.informer.com/7.2/). The sequences were further manually edited to exclude ambiguous portions to obtained non-structural ORF1 gene product YDR before proceeding for the final alignment. Multiple alignments for YDR sequences datasets were carried out using Clustal X2 Algorithm (http://www.clustal.org/clustal2/) [17]. The complete list of the sequences used for various host organisms are listed as additional files in the supplementary information (Additional file 1: S1 Table, Additional file 2: S2 Table, Additional file 3: S3 Table, Additional file 4: S4 Table, Additional file 5: S5 Table, Additional file 6: S6 Table, Additional file 7: S7 Table, Additional file 8: S8 Table).

### Nucleotide composition analysis

Nucleotide composition analysis of the YDR was calculated using MegaX software. The overall nucleotides occurrence frequency (A%, C%, T/U%, and G%), overall occurrence of nucleotide frequency at the third position of codon (A3%, C3%, U3%, and G3%) and overall occurrence of nucleotides frequencies of G+C at different codon positions were determined. The AUG and UGG codons were not considered for the analysis as they do not exhibit codon usage bias. The termination codons (UAG, UGA, UAA) were also excluded from the analysis since they do not encode any amino acid.

### Relative synonymous codon usage (RSCU) analysis

The ratio between the observed and expected usage frequency of a codon is described as the RSCU value if all synonymous codons are used equally for any specific amino acid [18]. The RSCU index was determined as follows:

$$RSCU=\frac{Gij}{\sum_j^{ni} Gij} ni$$

where *RSCU* is the relative synonymous codon usage value, *G*_*ij*_ is the observed number of the *i*th codon for the *j*th amino acid that has an “ni” type of synonymous codon. The RSCU values of the YDR were calculated using MegaX to determine the codon usage characteristics without the effect of amino acid composition and coding sequence length. Codons with RSCU values (> 1.6) and (< 0.6) were considered as “over-represented” and “under-represented” codons, respectively, whereas codons having the RSCU values (1) were regarded as not biased (average level codon). Moreover, less-abundant (RSCU < 1) and more-abundant (RSCU > 1) used codons were also determined.

### Relationship between overall nucleotide composition and nucleotide composition at the 3rd codon position

The correlation between A, T, G, C, GC, and 3rd codon position of its counterparts (A3, T3, G3, C3, GC3) were assessed. This was carried out to analyze whether if natural selection/mutation pressure individually contributed or if both collaboratively influenced the evolution of YDR in HEVs.

## Results

### Compositional features of YDR

**Fig. 1:**
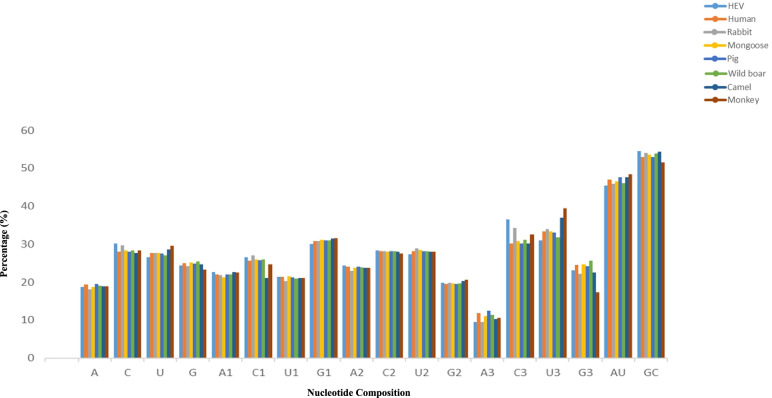
Comparative analysis of nucleotide composition patterns between HEV and its hosts (human, rabbit, mongoose, pig, wild boar, camel, and monkey)

The nucleotide composition values for YDR were calculated to analyze the effect of compositional constraints on codon usage (Table 1) (Fig. 1).

**Table 1:** Nucleotide composition analysis of YDR of hepatitis E viruses (%)

Nucleotide	HEV	Human	Rabbit	Mongoose	Pig	Wild boar	Camel	Monkey
**A**	18.841	19.319	18.127	18.705	19.532	19.108	18.910	18.960
**C**	30.169	28.022	29.816	28.287	28.048	28.391	27.662	28.287
**U**	26.631	27.654	27.777	27.777	27.485	27.014	28.671	29.510
**G**	24.357	25.003	24.277	25.229	24.933	25.485	24.755	23.241
**A1**	22.698	22.057	21.844	21.253	21.997	21.990	22.660	22.477
**C1**	26.665	25.688	27.013	25.993	25.792	25.908	21.100	24.770
**U1**	21.505	21.403	20.387	21.559	21.246	21.037	21.100	21.100
**G1**	30.131	30.851	30.784	31.192	30.963	31.062	31.467	31.651
**A2**	24.349	24.077	23.037	23.853	24.045	23.888	23.713	23.853
**C2**	28.387	28.186	28.134	27.981	28.175	28.158	27.941	27.522
**U2**	27.408	28.147	29.001	28.440	28.216	28.228	28.033	27.981
**G2**	19.853	19.588	19.826	19.724	19.562	19.724	20.312	20.642
**A3**	9.480	11.821	9.531	11.009	12.554	11.440	10.366	10.550
**C3**	36.452	30.193	34.301	30.886	30.177	31.108	30.275	32.568
**U3**	30.978	33.414	33.944	33.333	32.992	31.779	36.880	39.449
**G3**	23.088	24.570	22.222	24.770	24.275	25.670	22.477	17.431
**AU**	45.472	46.973	45.904	46.482	47.617	46.122	47.581	48.47
**GC**	54.526	53.025	54.093	53.516	52.981	53.876	54.417	51.528
**GC3**	59.54	54.763	56.523	55.656	54.452	56.778	52.752	49.999

The values are represented as percentage

#### HEV

The nucleotide composition trend was in order C > U > G > A, with an average of 30.169%, 26.631%, 24.357%, and 18.841%, respectively. Synonymous codons at the third position followed the trend C3S > U3S > G3S > A3S. The overall GC content was higher than that of AU, with 54.526% observed, compared with 45.472%, respectively, which indicates a GC-biased composition (Additional file 1: S1 Table).

#### Human

The nucleotide composition trend was in order C > U > G > A, with an average of 28.022%, 27.654%, 25.003%, and 19.319%, respectively. Synonymous codons at the third position followed the trend U3S > C3S > G3S > A3S. The overall GC content was higher than that of AU, with 53.025% observed, compared with 46.973%, respectively, which indicates a GC-biased composition (Additional file 2: S2 Table).

#### Rabbit

The nucleotide composition trend was in order C > U > G > A, with an average of 29.816%, 27.777%, 24.277%, and 18.127%, respectively. Synonymous codons at the third position followed the trend C3S > U3S > G3S > A3S. The overall GC content was higher than that of AU, with 54.093% observed, compared with 45.904%, respectively, which indicates a GC-biased composition (Additional file 3: S3 Table).

#### Mongoose

The nucleotide composition trend was in order C > U > G > A, with an average of 28.287%, 27.777%, 25.229%, and 18.705%, respectively. Synonymous codons at the third position followed the trend U3S > C3S > G3S > A3S. The overall GC content was higher than that of AU, with 53.516% observed, compared with 46.482%, respectively, which indicates a GC-biased composition (Additional file 4: S4 Table).

#### Pig

The nucleotide composition trend was in order C > U > G > A, with an average of 28.048%, 27.485%, 24.933%, and 19.532%, respectively. Synonymous codons at the third position followed the trend U3S > C3S > G3S > A3S. The overall GC content was higher than that of AU, with 52.981% observed, compared with 47.617% respectively, which indicates a GC-biased composition (Additional file 5: S5 Table).

#### Wild boar

The nucleotide composition trend in HEV was in order C > U > G > A, with an average of 28.391%, 27.014%, 25.485%, and 19.108%, respectively. Synonymous codons at the third position followed the trend U3S > C3S > G3S > A3S. The overall GC content was higher than that of AU, with 53.876% observed, compared with 46.122%, respectively, which indicates a GC-biased composition (Additional file 6: S6 Table).

#### Camel

The nucleotide composition trend in HEV was in order U > C > G > A, with an average of 28.671%, 27.662%, 24.755%, and 18.910%, respectively. Synonymous codons at the third position followed the trend U3S > C3S > G3S > A3S. The overall GC content was higher than that of AU, with 54.417% observed, compared with 47.581 respectively, which indicates a GC-biased composition (Additional file 7: S7 Table).

#### Monkey

The nucleotide composition trend in HEV was in order U > C > G > A, with an average of 29.510%, 28.287%, 23.241%, and 18.960%, respectively. Synonymous codons at the third position followed the trend U3S > C3S > G3S > A3S. The overall GC content was higher than that of AU, with 51.528% observed, compared with 48.47%, respectively, which indicates a GC-biased composition (Additional file 8: S8 Table).

Thus, the overall initial compositional findings revealed that YDR was richly endowed with C and U nucleotides. It was observed that the least chosen nucleotide in YDR was A. Moreover, the GC contents were significantly higher than that of AU contents (since AT content was <50%) in YDR.

### Patterns of codon usage in YDR

**Fig. 2:**
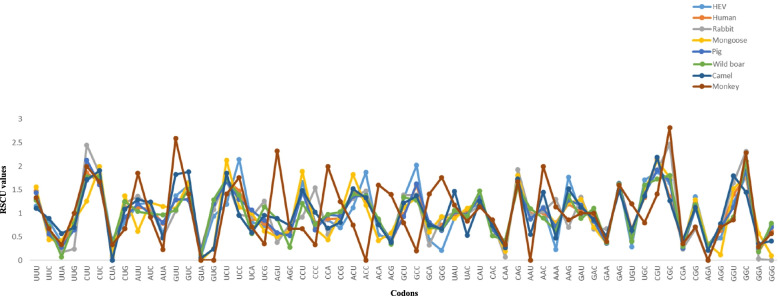
Comparative analysis of relative synonymous codon usage (RSCU) patterns between HEV and its hosts (human, rabbit, mongoose, pig, wild boar, camel, and monkey)

RSCU analysis was performed to assess the codon usage patterns and preferences for synonymous codons in the YDR. The RSCU values were computed for every codon in each gene sequence to decrypt the extent to which C/U-ended codons were preferred. The results are mentioned in Table 2 (Fig. 2).

**Table 2:** Average RSCU values of the codons of the HEV YDR and comparison with the RSCU values of its natural hosts

AA	Codon	HEV	Human	Rabbit	Mongoose	Pig	Wild boar	Camel	Monkey
**Phe (F)**	UUU	**1.16**	**1.48**	**1.3**	**1.56**	**1.44**	**1.28**	**1.11**	1.33
	UUC	**0.84**	0.52	**0.7**	0.44	0.56	0.72	**0.89**	0.67
**Leu (L)**	UUA	0.39	0.28	0.17	0.42	0.28	0.07	0.57	0.33
	UUG	0.81	0.77	0.24	0.84	0.65	0.79	0.7	1
	CUU	**1.7**	**1.86**	**2.45**	**1.26**	**2.13**	**1.78**	**1.72**	**2**
	CUC	**1.91**	**1.66**	**1.84**	**2**	**1.61**	**1.78**	**1.91**	**1.67**
	CUA	0.37	0.29	0.07	0.11	0.42	0.34	0	0.33
	CUG	0.81	**1.13**	**1.23**	**1.37**	0.92	**1.25**	1.09	0.67
**Ile (I)**	AUU	**1.19**	**1.12**	**1.36**	0.62	**1.2**	**1.04**	**1.29**	**1.85**
	AUC	**1.24**	**1.09**	**1.15**	**1.23**	**0.99**	**0.99**	**1.24**	**0.92**
	AUA	0.57	0.79	0.5	**1.15**	0.81	**0.97**	0.48	0.23
**Val (V)**	GUU	**1.37**	**1.29**	**1.05**	**1.1**	**1.29**	**1.07**	**1.83**	**2.59**
	GUC	**1.59**	**1.3**	**1.66**	**1.65**	**1.29**	**1.51**	**1.88**	**1.41**
	GUA	0.1	0.25	0.22	0	0.22	0.13	0.05	0
	GUG	**0.94**	**1.17**	**1.08**	**0.25**	**1.2**	**1.29**	0.24	0
**Ser (S)**	UCU	1.19	**1.72**	**1.72**	**2.13**	**1.67**	**1.73**	**1.85**	**1.41**
	UCC	**2.15**	**1.48**	0.97	1.13	1.3	**1.39**	0.96	**1.76**
	UCA	0.81	0.89	0.92	1	1.07	0.57	0.59	0.71
	UCG	0.74	0.79	1.26	0.63	0.85	1.14	0.96	0.35
	AGU	0.59	0.51	0.38	0.5	0.58	0.88	0.89	**2.33**
	AGC	0.52	0.6	0.76	0.63	0.53	0.28	0.74	0.67
**Pro (P)**	CCU	**1.66**	**1.44**	0.92	**1.89**	**1.45**	1.21	**1.49**	0.67
	CCC	0.8	0.78	**1.54**	0.67	0.65	0.77	1.02	0.33
	CCA	0.86	0.88	0.55	0.44	0.97	0.98	0.68	**2**
	CCG	0.69	0.9	0.99	1	0.94	1.03	0.81	**1.25**
**Thr (T)**	ACU	**1.12**	**1.5**	**1.32**	**1.83**	**1.36**	**1.41**	**1.52**	0.75
	ACC	**1.87**	**1.27**	**1.48**	**1.17**	**1.38**	**1.37**	**1.33**	0
	ACA	0.54	0.85	0.77	0.42	0.84	0.88	0.76	**1.6**
	ACG	0.47	0.38	0.42	0.58	0.42	0.34	0.38	**1.4**
**Ala (A)**	GCU	**1.33**	**0.98**	**1.39**	**1.13**	**0.93**	**1.34**	**1.22**	**0.8**
	GCC	**2.02**	**1.59**	**1.39**	**1.33**	**1.63**	**1.28**	**1.37**	0.2
	GCA	0.43	0.68	0.32	0.6	**0.81**	0.71	**0.75**	**1.41**
	GCG	0.21	**0.75**	**0.91**	**0.93**	0.64	0.66	0.67	**1.76**
**Tyr (Y)**	UAU	**0.91**	**0.99**	**1.02**	**0.89**	**1.03**	**1.06**	**1.47**	**1.17**
	UAC	**1.09**	**1.01**	**0.98**	**1.11**	**0.97**	**0.94**	0.53	**0.83**
**His (H)**	CAU	**1.2**	**1.34**	**1.17**	**1.24**	**1.34**	**1.48**	**1.26**	**1.14**
	CAC	0.8	0.66	0.83	0.76	0.66	0.52	0.74	0.86
**Gln (Q)**	CAA	0.36	0.27	0.07	0.19	0.31	0.43	0.27	0.33
	CAG	**1.64**	1.73	**1.93**	**1.81**	**1.69**	**1.57**	**1.73**	**1.67**
**Arn (N)**	AAU	0.87	0.99	0.96	0.89	0.89	1.1	0.55	0
	AAC	1.13	1.01	1.04	1.11	1.11	0.9	1.45	2
**Lys (K)**	AAA	0.23	0.8	**1.3**	0.78	0.72	0.63	0.48	**1.14**
	AAG	**1.77**	**1.2**	0.7	**1.22**	**1.28**	**1.38**	**1.52**	0.86
**Asp (D)**	GAU	**0.98**	**1.03**	**1.34**	**1.3**	**1.17**	0.89	**1.13**	**1**
	GAC	**1.02**	**0.97**	0.66	0.7	0.83	**1.11**	0.87	**1**
**Glu (E)**	GAA	0.37	0.38	0.67	0.36	0.36	0.38	0.52	0.4
	GAG	**1.63**	**1.62**	**1.33**	**1.64**	**1.64**	**1.62**	**1.48**	**1.6**
**Cys (C)**	UGU	0.29	0.67	0.43	0.53	0.59	0.4	0.64	1.2
	UGC	**1.71**	1.33	**1.57**	**1.47**	1.41	**1.6**	1.36	0.8
**Arg (R)**	CGU	**1.88**	**2.16**	**1.92**	**2.12**	**1.92**	**1.73**	**2.19**	**1.41**
	CGC	**1.65**	**1.35**	**2.47**	**1.76**	1.7	**1.81**	1.27	**2.82**
	CGA	0.3	0.34	0.24	0.35	0.26	0.3	0.42	0.35
	CGG	**1.35**	1.22	0.67	**1.29**	1.1	1.18	1.13	0.71
	AGA	0.34	0.3	0	0.35	0.31	0.3	0.21	0
	AGG	0.48	0.64	0.71	0.12	0.7	0.68	0.78	0.71
**Gly (G)**	GGU	**1.24**	**1.4**	**1.65**	**1.52**	**1.13**	0.92	**1.8**	0.86
	GGC	**1.79**	**1.8**	**2.32**	**1.81**	**1.91**	**2.11**	**1.45**	**2.29**
	GGA	0.23	0.19	0.03	0.57	0.26	0.18	0.35	0.29
	GGG	0.74	0.62	0	0.1	0.7	0.79	0.41	0.57

The preferred codons are indicated in bold

#### HEV

Among the 29 preferred codons, 24 were U/C-ending (U-ending: 12; C-ending: 12;) and 5 were G/A-ending (G-ending: 5; A-ending: 0) (Table 2) (Additional file 9: S9 Table). This result inferred that U- and C-ending codons are preferred in coding sequences. Within these preferred codons, 13 had a RSCU value >1.6, i.e., overrepresented codons (CUU, CUC, UCC, CCU, ACC, GCC, CAG, AAG, GAG, UGC, CGU, CGC, GGC), while the remaining 16 had RSCU values >0.6 and <1.6. Out of these 16, 4 codons had RSCU values <1, i.e., less-abundant codons (UUC, GUG UAU, GAU), and 12 had RSCU values >1, i.e., abundant codons (UUU, AUU, AUC, GUU, GUC, ACU, GCU, UAC, CAU, GAC, CGG, GGU). No optional synonymous codons were underrepresented (RSCU < 0.6).

#### Human

Among the 28 preferred codons, 23 were U/C-ending (U-ending: 13; C-ending: 10;) and 5 were G/A-ending (G-ending: 5; A-ending: 0) (Table 2) (Additional file 10: S10 Table). This result inferred that U- and C-ending codons are preferred in coding sequences. Within these preferred codons, 6 had a RSCU value >1.6, i.e., overrepresented codons (CUU, CUC, UCU, GAG, CGU, GGC), while the remaining 22 had RSCU values >0.6 and <1.6. Out of these 22, 4 codons had RSCU values <1, i.e., less-abundant codons (GCU, GCG, UAU, GAC), while 18 had RSCU values >1, i.e., abundant codons (UUU, CUG, AUU, AUC, GUU, GUC, GUG, UCC, CCU, ACU, ACC, GCC, UAC, CAU, AAG, GAU, CGC, GGU). No optional synonymous codons were underrepresented (RSCU < 0.6).

#### Rabbit

Among the 29 preferred codons, 23 were U/C-ending (U-ending: 12; C-ending: 11) and 6 were G/A-ending (G-ending: 5; A-ending: 1) (Table 2) (Additional file 11: S11 Table). This result inferred that U- and C-ending codons are preferred in coding sequences. Within these preferred codons, 7 had RSCU value >1.6, i.e., overrepresented codons (CUU, CUC, GUC, UCU, CAG, CGU, CGC), while the remaining 22 preferred codons had RSCU values >0.6 and <1.6. Out of these 22, 3 codons had RSCU values <1, i.e., less-abundant codons (UUU, GCG, UAC), while 19 had RSCU values >1, i.e., abundant codons (UUC, CUG, AUU, AUC, GUU, GUG, CCC, ACU, ACC, GCU, GCC, UAU, CAU, AAA, GAU, GAG, UGC, GGU, GGC). No optional synonymous codons were underrepresented (RSCU < 0.6).

#### Mongoose

Among the 29 preferred codons, 21 were U/C-ending (U-ending: 12; C-ending: 9) and 8 were G/A -ending (G-ending: 7; A-ending: 1) (Table 2) (Additional file 12: S12 Table). This result inferred that U-and C-ending codons are preferred in coding sequences. Within these preferred codons, 10 had RSCU value >1.6, i.e., overrepresented codons (CUC, GUC, UCU, CCU, ACU, CAG, GAG, CGU, CGC, GGC) while the remaining 19 preferred codons had RSCU values >0.6 and <1.6. Out of these 19, 3 codons had RSCU values <1, i.e., less-abundant codons (GUG, GCG, UAC), while 16 had RSCU values >1, i.e., abundant codons (UUU, CUU, CUG, AUC, AUA, GUU, ACC, GCU, GCC, UAU, CAU, AAG, GAU, UGC, CGG, GGU). One optional synonymous codon was underrepresented (RSCU < 0.6).

#### Pig

Among the 25 preferred codons, 20 preferred codons were U/C-ending (U-ending: 13; C-ending: 7) and 6 were G/A -ending (G-ending: 4; A-ending: (1) (Table 1) (Additional file 13: S13 Table). This result inferred that U-and C-ending are preferred in coding sequences. Within these preferred codons, 8 had RSCU value >1.6, i.e., overrepresented codons (CUU, CUC, UCU, GCC, CAG, GAG, CGU, GGC), while the remaining 17 preferred codons had RSCU values >0.6 and <1.6. Out of these 17, 4 codons had RSCU values <1, i.e., less-abundant codons (AUC, GCU, GCA, UAC), while 13 had RSCU values >1, i.e., abundant codons (UUU, AUU, GUU, GUC, GUG, CCU, ACU, ACC, UAU, CAU, AAG, GAU, GGU). No optional synonymous codon was underrepresented (RSCU < 0.6).

#### Wild boar

Among the 27 preferred codons, 21 preferred codons were C/U-ending (C-ending: 11; U-ending: 10) and 6 were G/A -ending (G-ending: 5; A-ending: 1) (Table 1) (Additional file 14: S14 Table). This result inferred that C- and U-ending are preferred in YDR. Within these preferred codons, 8 had RSCU value >1.6, i.e., overrepresented codons (CUU, CUC, UCU, GAG, UGC, CGU, CGC, GGC), while the remaining 19 preferred codons had RSCU values >0.6 and <1.6. Out of these 19, 3 codons had RSCU values <1, i.e., less-abundant codons (AUC, AUA, UAC), while 16 had RSCU values >1, i.e., abundant codons (UUU, CUG, AUU, GUU, GUC, GUG, UCC, ACU, ACC, GCU, GCC, UAU, CAU, CAG, AAG, GAC). No optional synonymous codon was underrepresented (RSCU < 0.6).

#### Camel

Among the 24 preferred codons, 20 preferred codons were U/C-ending (U-ending: 13; C-ending: 7) and 4 were G/A -ending (G-ending: 3; A-ending: 1) (Table 2) (Additional file 15: S15 Table). This result inferred that U- and C-ending are preferred in YDR. Within these preferred codons, 8 had RSCU value >1.6, i.e., overrepresented codons (CUU, CUC, GUU, GUC, UCU, CAG, CGU, GGU), while the remaining 16 preferred codons had RSCU values >0.6 and <1.6. Out of these 16, 2 codons had RSCU values <1, i.e., less-abundant codons (UUC, GCA), while 14 had RSCU values >1, i.e., abundant codons (UUU, AUU, AUC, CCU, ACU, ACC, GCU, GCC, UAU, CAU, AAG, GAU, GAG GGC). No optional synonymous codon was underrepresented (RSCU < 0.6).

#### Monkey

Among the 27 preferred codons, 18 preferred codons were U/C-ending (U-ending: 10; C-ending: 8) and 9 were G/A -ending (G-ending: 5; A-ending: 4) (Table 2) (Additional file 16: S16 Table). This result inferred that U- and C-ending are preferred in YDR. Within these preferred codons, 13 had RSCU value >1.6, i.e., overrepresented codons (CUU, CUC, AUU, GUU, GAG, UCC, AGU, CCA, GCG, CAG, GAG, CGC, GGC), while the remaining 14 preferred codons had RSCU values >0.6 and <1.6. Out of these 14, 3 codons had RSCU values <1, i.e., less-abundant codons (AUC, UAC, GCU), while 9 had RSCU values >1, i.e., abundant codons (GUC, UCU, CCG, ACG, GCA, UAU, CAU, AAA, CGU). In addition to this, 2 had RSCU values 1, i.e., random codons (GAU, GAC). No optional synonymous codon was underrepresented (RSCU < 0.6).

In line with compositional analysis, the RSCU analysis confirmed the codon biasness towards U- and C-ended codons. The RSCU pattern clearly indicated that the selection of preferred codons showed common attributes as well as differences among HEV and HEV-hosts (Table 2). It was observed that some of the codons showed similar preference among HEV and HEV-hosts, while for other codons, HEV showed preference differed from that of its hosts or vice-versa. Thus, the codon which is most common among HEV and HEV-hosts, is considered as the most preferred codon that codes for a particular amino acid. Because the optimal codon selection in viruses largely depends on their hosts, we next compared the codon usage frequency of HEV with its hosts by correlating their RSCU patterns.

### Relationship among HEV-hosts by comparing codon usage frequency

Since a particular amino acid is encoded by a preferred codon, the usage of synonymous codons is not random. Thus, we calculated the frequency of the preferred codons for each amino acid using the RSCU analysis (Additional file 9: S9 Table, Additional file 10: S10 Table, Additional file 11: S11 Table, Additional file 12: S12 Table, Additional file 13: S13 Table, Additional file 14: S14 Table, Additional file 15: S15 Table and Additional file 16: S16 Table), to analyze the relationship among HEV and its hosts. This was done to understand the influence of selection pressure from hosts on codon usage patterns of HEV. A list of preferred codons encoding amino acids with higher frequency as compared to other synonymous codons for HEV, and all the hosts were computed and compared as mentioned in Table 3.

**Table 3:**
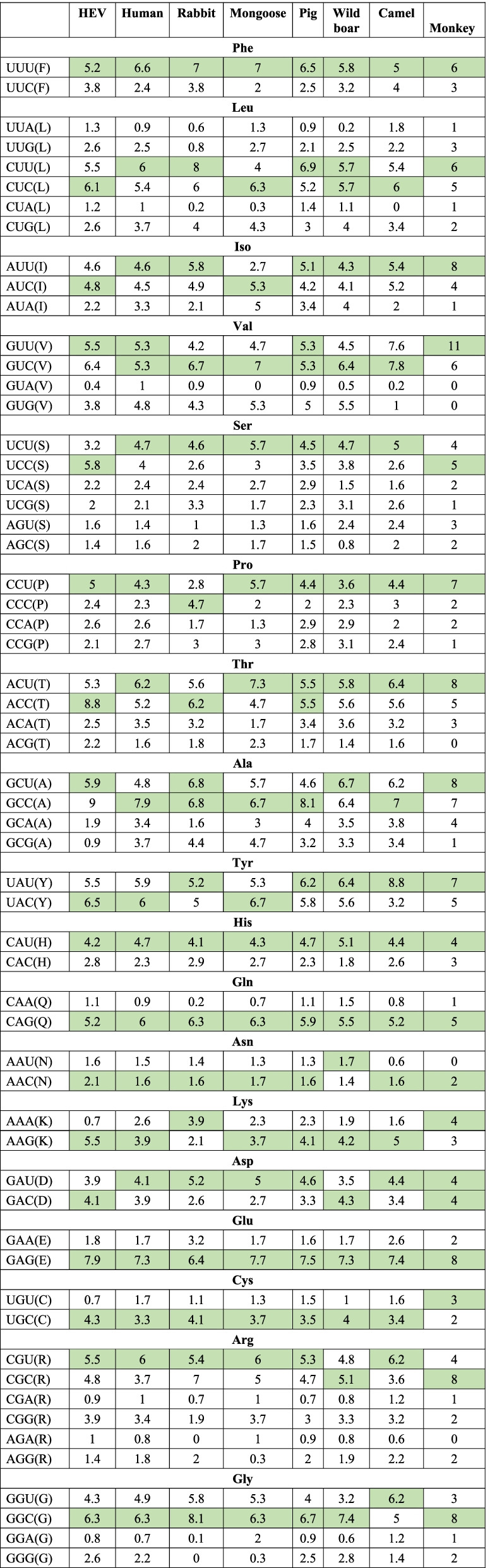
Preferred codons for each amino acid in the YDR of HEV and its hosts

Comparison of codon usage frequency of preferred codons among HEV and its hosts. All the preferred codons are highlighted indicating the highest codon frequency. Thus, codon usage pattern of YDR was a mix of coincidence and antagonism with respect to its host

The observed 4 amino acids Phe, His, Gln, and Glu showed similar usage of preferred codons (UUU, CAU, CAG, and GAG) among HEV and its hosts, which implicates an evidence of mutual codon preference. While few amino acids also showed differences in their choice of preferred codons. HEV and other HEV-hosts (human, rabbit, mongoose, pig, wild boar, camel) shared evidence of preferred codons (GUC, UGC, and CGU) for encoding the amino acids Val, Cys, and Arg, respectively, except for monkey which used different set of preferred codons (GUU, UGU, and CGC). Moreover, this phenomenon was also observed in other hosts, i.e., preferred codons encoding amino acids was different in specific host in comparison to other HEV-hosts and HEV. Firstly, HEV and HEV-hosts (human, mongoose, pig, wild boar, camel, and monkey) shared evidence of preferred codon for CCU which encoded Pro, except for rabbit which preferred CCC over CCU. Secondly, HEV and HEV-hosts (human, mongoose, rabbit, pig, camel, and monkey) shared evidence of preferred codon for AAC for encoding Asn, except for wild boar, which preferred AAU over AAC. Thirdly, HEV and HEV-hosts (human, mongoose, rabbit, pig, wild boar, and monkey) shared evidence of preferred codon for GGC for encoding Gly, except for camel which preferred GGU over GGC (Table 3).

In detail, among the 18 preferred codons in HEV, 13 were common between HEV and human; 11 were common between HEV and rabbit; 15 were common between HEV and mongoose; 13 were common between HEV and pig; 13 were common between HEV and wild boar; 12 were common between HEV and camel; and 8 were common between HEV and monkey (Table 3). Therefore, the abovementioned codons were common between HEV and respective hosts, indicating coincident codon usage portion, i.e., these preferred codons were commonly shared between the virus and host. However, discrepancies were also observed within the preferred codons between HEV and its hosts, i.e., dissimilar usage of preferred codons. Thus, the ratio of coincident/antagonist preferred codons was 13/5 between HEV and human; 11/7 between HEV and rabbit; 15/3 between HEV and mongoose; 13/5 between HEV and pig; 13/5 between HEV and wild boar; 12/6 between HEV and camel; and 8/10 between HEV and monkey. Thus, codon usage pattern of HEV YDR is a mix of coincidence and antagonism with respect to its hosts.

Thus, for a particular amino acid, if a preferred codon in HEV showed similarity with its host cell, this phenomenon is termed as “mutual codon preference of host–pathogens”. This implies that similar codon usage pattern among HEV and HEV-hosts could help the virus to synthesize the amino acid and corresponding proteins in a more efficient manner, thus helping the pathogen to thrive in its host cells. On the contrary, the difference in preferred codon among HEV and HEV-hosts suggests lack of shared codon preference, causing reduction in the translation efficiency of the corresponding amino acids.

**Fig. 3:**
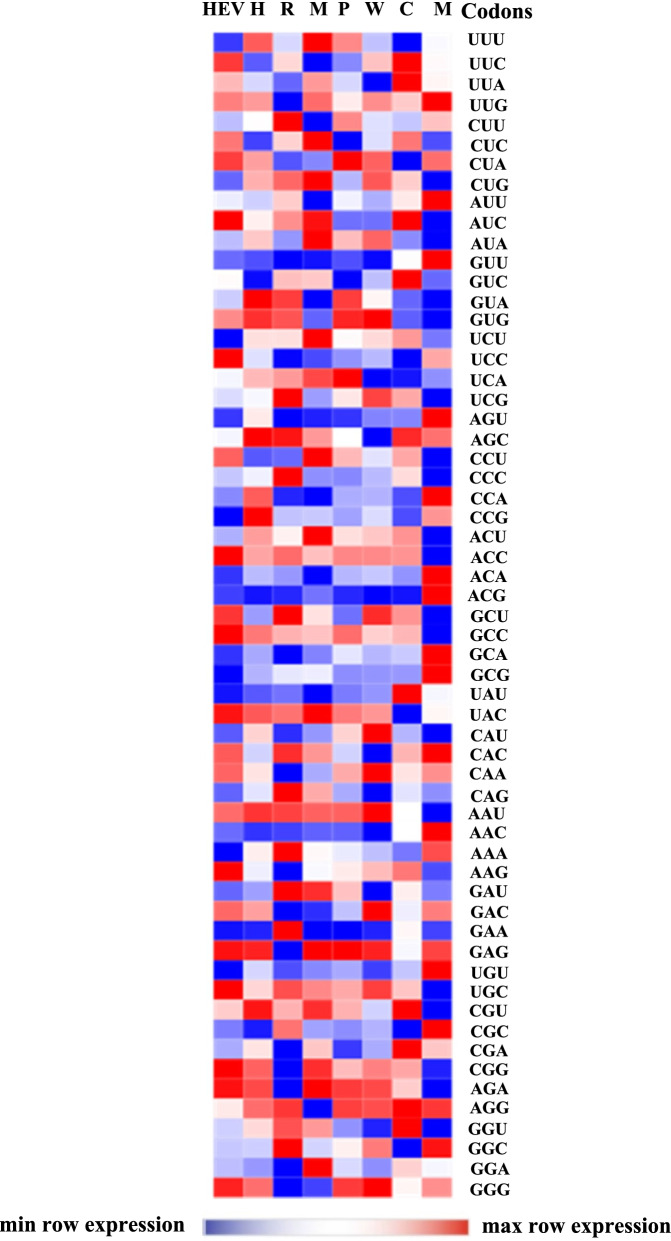
Heat map showing the relative synonymous codon usage (RSCU) values accompanying different hosts (H: human, R: rabbit, M: mongoose, P: pig, W: wild boar, C: camel, and M: monkey). The host species are mentioned on the horizontal axis and codons are represented on the vertical axis. Heatmap confirms the occurrence of resemblance as well as discrepancies in RSCU pattern among different hosts

A heat map was constructed using RSCU values of various HEV strains and its hosts (Fig. 3), which revealed that HEV codon preference neither completely differed nor completely showed similarity with its hosts, indicating a mixture of similar and dissimilar codon preferences (Fig. 3). Moreover, the top five most and least frequent used codons were also identified which showed common attributes and differences in codon usage patterns of HEV isolates (Table 4).

**Table 4:** Most frequent and least used codons among HEV and its natural hosts

**Top 5 most frequent used codons**	
**HEV**	ACC (8.8), GAG (7.9), UAC (6.5), GUC (6.4), GGC (6.3),
**Human**	GCC (7.9), UUU (6.6), GGC (6.3), ACU (6.2), CGU (6.0)
**Rabbit**	GGC (8.1), CUU (8.0), CGC/UUU (7.0), GCU/GCC (6.8), GUC (6.7)
**Mongoose**	GAG (7.7), ACU (7.3), UUU/GUC (7.0), GCC/UAC (6.7), GGC/CAG/CUC (6.3)
**Pig**	GCC (8.1), GAG (7.5), CUU (6.9), GGC (6.7), UUU (6.5)
**WB**	GGC (7.4), GAG (7.3), GCU (6.7), GUC/GCC/UAU (6.4), UUU/ACU (5.8)
**Camel**	UAU (8.8), GUC (7.8), GUU (7.6), GAG (7.4), GCC (7.0)
**Monkey**	GUU (11), AUU/ACU/GCU/GAG/CGC/GGC (8), CCU/GCC/UAU (7), UUU/CUU/GUC (6), CUC/UCC/ACC/UAC/CAG (5)
**Top 5 least frequent used codons**	
**HEV**	GUA (0.4), UGU/AAA (0.7), GGA (0.8), CGA/GCG (0.9), AGA (1)
**Human**	GGA (0.7), AGA (0.8), CAA/UUA (0.9), CUA/GUA/CGA (1.0), AGU (1.4)
**Rabbit**	AGA/GGG (0), GGA (0.1), CUA/CAA (0.2), UUA (0.6), CGA (0.7), UUG (0.8)
**Mongoose**	GUA (0), CUA/GGG/AGG (0.3), CAA (0.7), AGA/CGA (1.0), UUA/CCA/AGU/UGU/AAU (1.3), UCG/ACA/AAC/GAA/AGC (1.7),
**Pig**	CGA (0.7), UUA/GUA/AGA/GGA (0.9), CAA (1.1), AAU (1.3), CUA (1.4)
**Wild boar**	UUA (0.2), GUA (0.5), GGA (0.6), CGA/AGC/AGA (0.8), UGU (1.0)
**Camel**	CUA (0), GUA (0.2), AAU/AGA (0.6), CAA (0.8), GUG (1.0), CGA/GGA (1.2)
**Monkey**	GUA/GUG/ACG/AAU/AGA (0), UUA/CUA/AUAUCG/CCG/GCG/CAA/CGA/GGA (1), CUG/UCA/CCC/CCA/AAC/GAA/UGC/CGG/AGC/AGG/GGG (2), UUC/UUG/ACA/CAC/AAG/UGU/AGU/GGU (3), AUC/UCU/GCA/CAU/AAA/GAU/GAC/CGU (4), CUC/UCC/ACC/UAC/CAG (5)

Codon frequency is given in parentheses following the relative synonymous codon usage

Thus, our results clearly indicated that the synonymous codon usage of HEV is a mixture of the two types of codon usage: “coincidence and antagonism.”

### Effect of natural selection in shaping codon usage patterns

It has been suggested that the frequencies of nucleotides A and U /T should be equal to that of C and G at the third position of the codon if mutational pressure affects the synonymous codon usage bias [28]. However, huge variations were noted in the nucleotide base composition in case of all the hosts, signifying that synonymous codon usage bias could majorly be influenced by natural selection (Table 1). From these findings, it was clear that compositional constraints under mutation pressure combined with natural selection shaped the HEV YDR across all its hosts.

## Discussion

Inspection of factors governing protein evolution is essential for various research fields, including comparative genomics, molecular evolution, and structural biology. With this study, we implemented a systematic survey of the evolutionary pressures (i.e., mutational bias and natural selection) across the YDR to gain insights into the HEV functional implications in regulation as well as adaptative evolution.

Jenkins and Holmes (2003) reported that codon usage bias phenomenon can be influenced by the overall nucleotide composition pattern [37]. Thus, initially, we computed the nucleotide frequencies of the YDR from HEV and its hosts. The HEV YDR revealed an over-representation of C, with overall C/U codon bias pattern in the nucleotide composition. In HEV, the percentage of C was the highest followed by U and G, with A having the lowest value (except hosts, camel, and monkey which followed the trend U > C > G > A). This clearly revealed that there was unequal distribution of A, U, G, and C nucleotides among the YDR codons. Additionally, in HEV and rabbit, the nucleotide values at third codon positions also followed the same trend, i.e., C3 had the highest value, followed by U3, G3, and A3 with the least value (while hosts followed the trend C3 > U3 > G3 > A3). Therefore, it could be interpreted that the initial nucleotide compositional patterns showed more preference towards C- and U-ended codons followed by G/A-ended codons. This is consistent with the recent investigation that has reported U/C rich genome in ORF1 of HEV [45]. However, the overall C/U rich pattern in the nucleotide content in YDR is opposite to the pattern observed in RNA viruses, which showed the prevalence of A/C-rich genomes (HIV, hepatitis C, rubella viruses) [46]. Thus, it could be interpreted that this biasness in YDR was due to the adaptation of common ancestor of modern HEV strains in terms of nucleotide composition requirement of the host during its process of evolution [47].

It has been suggested that particularly in viruses, AU- or GC-rich genomes tends to correlate with the RSCU patterns. For instance, AU- or GC-rich composition preferred codons ending with either A and U or G and C, respectively. These trends, when observed, support the influence of mutational pressure [37]. The RSCU analysis revealed that HEV had comparatively higher codon usage bias towards U- and C-ended codons. The overall RSCU patterns can potentially hide host-specific patterns, so we next calculated the RSCU values for specific hosts. Thus, the comparative analysis was performed among HEV and its hosts, by correlating their RSCU patterns. It was noted that the host-specific codon usage patterns also showed preferred codons ending with U and C. Thus, in line with nucleotide composition analysis, the RSCU analysis further confirmed the codon biasness towards U- and C-ended codons. Thus, it could be interpreted that mutational bias was found to be a major force determining the codon usage patterns of YDR, which probably suggested that compositional constraints influenced the selection of preferred codons. However, it is interesting to mention that though HEV and its hosts was endowed with higher percentage of GC rather than AC, the RSCU analysis revealed a biasness towards U-terminated codons. This suggested that other factors in combination with mutation pressure also existed in the process of HEV evolution. Therefore, selection pressure from hosts contributed to shaping the molecular evolution of HEV at the level of codon usage.

The codon usage in virus’s genome in accordance with its host codon preferences is an important aspect which determines the evolutionary adaptation of the virus to its host cell. The alteration of codon usage in viral genomes due to the proper information obtained from host genes regulates the virus-host interactions [48]. As viruses are obligate parasites, their optimal codon selection is largely dependent on their host cells translational machinery [49]. A noteworthy variation was observed in the usage for the preferred codons among HEV and HEV-hosts. This implied that the codon usage patterns of HEV as well as the possible fitness of HEV to adapt within its dynamic host range were largely influenced by the selection pressures exerted from HEV-hosts.

In this study, it was observed, that unlike other viruses, that have evolved completely identical to their hosts or completely opposite to their hosts codon patterns [50, 51], the HEV evolution showed a mixture of two codon usage patterns. Our results revealed that none of the hosts showed complete resemblance or complete discrepancy to the HEV. The ratio of common/uncommon preferred codons between HEV-Human, HEV-Rabbit, HEV-Mongoose, HEV-Pig, HEV-Wild boar, HEV-Camel, and HEV-Monkey were 13/5, 11/7, 15/3, 13/5, 13/5, 12/6, and 8/10, respectively. Thus, codon usage pattern of HEV YDR showed a mixture of coincidence and antagonism with respect to its hosts. The resemblance in synonymous codon patterns among HEV and its hosts implied that HEV could adapt to its host cells, resulting in its multiplication. This phenomenon suggests that the virus can replicate in host cells due to similarity in usage for preferred codons. It has been suggested that the coincident portions of codon usage could facilitate efficient translation of the corresponding amino acids among viruses and their respective hosts [52]. This indicates that preferred codons or more abundant tRNA molecules are chosen to increase the accuracy of translation [53]. While the antagonistic portions of codon usage may aid in proper folding of viral proteins, even though decrease in the corresponding amino acids translation efficiency is observed [52]. This implies that rare codons help in reducing the inappropriate co-translational folding of proteins [54]. Thus, our results clearly suggested that the codon usage pattern of HEV is both coincident and antagonistic to that of its hosts. Such patterns of coincidence and antagonism have been previously reported in HBV [55], HCV [52] and enterovirus 71 [29]. Therefore, our results probably suggested that disfavored codons encoding amino acids cannot be considered as a deleterious factor for viral genes in order to adapt to its hosts.

Thus, it could be interpreted that the influence of compositional constraints, codon usage biasness, and mutational alongside the selective forces were reflected in the occurrence of HEV YDR codon usage patterns.

## Conclusions

To the best of knowledge, this report documents the codon usage analysis in HEV YDR for the first time using bioinformatics approach. Thisnovel approach is expected to strengthen our understanding on the common attributes and differences in the codon usage patterns among HEV and its various hosts. The nucleotide compositional analysis showed relative abundance of C and U nucleotides and relative synonymous codon usage analysis revealed that the preferred synonymous codons mostly end with C/U. Moreover, it was observed that the HEV codon usage pattern to that of its host cells is a mixture of coincidence and antagonism. The compositional characteristics indicated that interaction between the mutation pressure from virus and translation selection from host exist in the processes of HEV evolution. Our study suggested that synonymous codon usage in HEV is an evolutionary process, perhaps reflecting a dynamic process of mutation and selection forces to adjust its codon usage to different hosts and conditions. The present study is thus envisaged to infer the evolution, adaptation, and biology of HEV via specific codon preferences.

## Supplementary Information

**Additional file 1.**

**Additional file 2.**

**Additional file 3.**

**Additional file 4.**

**Additional file 5.**

**Additional file 6.**

**Additional file 7.**

**Additional file 8.**

**Additional file 9.**

**Additional file 10.**

**Additional file 11.**

**Additional file 12.**

**Additional file 13.**

**Additional file 14.**

**Additional file 15.**

**Additional file 16.**

## Data Availability

Not applicable.

## Abbreviations

HEV: Hepatitis E virus
YDR: Y-domain region
RSCU: Relative synonymous codon usage
